# Maternal Satisfaction With Children's Vaccination and Its Contributing Factors in Ethiopia: A Systematic Review and Meta-Analysis

**DOI:** 10.1155/2024/4213025

**Published:** 2024-10-05

**Authors:** Nega Tezera Assimamaw, Aklilu Endalamaw, Mengistu Makonnen Kelkay, Almaz Tefera Gonete, Bewuketu Terefe, Kassaye Ahmed Zeleke

**Affiliations:** ^1^Department of Pediatrics and Child Health Nursing, School of Nursing, College of Medicine and Health Sciences, University of Gondar, Gondar, Ethiopia; ^2^School of Public Health, The University of Queensland, Brisbane, Australia; ^3^Department of Community Health Nursing, School of Nursing, College of Medicine and Health Sciences, University of Gondar, Gondar, Ethiopia; ^4^Department of Neonatal Nursing, School of Nursing, College of Medicine and Health Sciences, University of Gondar, Gondar, Ethiopia

**Keywords:** children's vaccination, Ethiopia, maternal satisfaction, systematic study and meta-analysis

## Abstract

**Background:** Various initiatives are underway to improve maternal satisfaction with the vaccination of children in developing nations. Governments, international organizations, and nongovernmental organizations are actively working to improve healthcare infrastructure, expand service accessibility, improve communication, and foster community engagement. However, despite these efforts, maternal satisfaction with child vaccination services continues to be a significant issue.

**Objective:** This systematic review and meta-analysis is aimed at assessing the pooled prevalence of maternal satisfaction with the child's vaccination service and its predictors in Ethiopia.

**Methods:** Scopus, Embase, Web of Science, Google Scholar, PubMed, African Journals Online, and Semantic Scholar were searched to access the included articles. A weighted inverse-variance random effect model was used to estimate the prevalence of maternal satisfaction with vaccination of children. Variations in pooled prevalence estimates were adjusted by subgroup analysis according to the specific region where the study was conducted. Funnel plot and Egger's regression test were used to check publication bias. STATA version 14 statistical software was used for meta-analysis.

**Results:** The combined prevalence of maternal satisfaction with vaccination of children was found to be 73% (95% CI: 72–75; *I*^2^ = 0.00%, *p* value < 0.001). Based on the subgroup analysis, the result revealed that the prevalence of maternal satisfaction with vaccination of children was 63% in SNNPR, 79% in Oromia, and 74% in Amhara.

**Conclusions:** A meta-analysis of mothers' satisfaction with vaccination services for their children in Ethiopia found a low level of satisfaction. Therefore, provide regular training and capacity-building programs for healthcare workers involved in the delivery of vaccination services.

## 1. Introduction

A satisfactory level of immunization is an essential component of the expanded program on the delivery of vaccine services. Satisfaction is, therefore, defined as the degree of satisfaction of the service in enhancing the coverage and quality of child vaccination [1–3].

Worldwide, the expanded program of vaccination has successfully prevented more than two million child infectious disease-related deaths annually [4, 5]. To increase vaccination rates and achieve 100% coverage by 1990, Ethiopia has been implementing the Expanded Immunization Program since 1980 [6].

Despite numerous interventions, the results of the umbrella review indicated that 57.72% of Ethiopian children aged 12–23 months received full immunization coverage. It falls below the target set by the World Health Organization (WHO), which aims for immunization coverage rates of more than 90% [7].

Significant interventions included the development of basic healthcare services, the installation of an inclusive health extension package, and the training of front-line health extension staff throughout the nation [4, 8].

Maternal satisfaction measures the quality of care at the policy, service delivery, and client levels using a variety of indicators, including the chosen method of service, technical proficiency and information provided to clients, social interactions, follow-up and consistency mechanisms, and the appropriate combination of services [9, 10].

However, it is still not met even now due to a number of variables, including low parental understanding of infant immunization, a shortage of trained professionals, inaccessibility to services, poor health facilities, high rates of abandonment, a lack of financial support, and a significant staff turnover rate [6, 8, 11].

The main obstacles to satisfaction with vaccinations are misunderstanding, a mother's urge to breastfeed the infant while inoculated, and an inadequate level of professional care about childhood vaccination [12].

Based on various research findings, mother satisfaction is a vital factor in raising the excellence of EPI services, which will subsequently improve overall utilization. The level of customer satisfaction with childhood immunization services was largely influenced by the facility opening time, the length of time patients had to wait for immunization services, the service supplier's method, and the location in which services were delivered [13–19].

Consequently, assessing clients' satisfaction with the immunization service is functionally significant because satisfied clients are more likely to adhere to intervention and return to the childhood immunization facility. It will additionally facilitate in spreading positive vibes to others, being proactive in their own services, persisting to use healthcare amenities, and mentioning the facility's services to everyone else. Clients who are dissatisfied with a service, on the contrary, are less likely to return or use immunization services, resulting in a main determinant of children receiving less than acceptable vaccination in underdeveloped and developing nations [20–23].

Previously, several studies attempted to quantify the level of maternal satisfaction with children's vaccination services in Ethiopia on an individual basis [1, 9, 12, 14, 24–27]. However, there was no comprehensive review in this area. Hence, the objective of this systematic review is to quantify the summarized prevalence level of maternal satisfaction with the standard of child vaccination services offered in Ethiopia.

Therefore, the results of this study will assist decision makers, child healthcare workers, and responsible parties in assessing mothers' satisfaction with child immunization at the national level, with the intent of decreasing dissatisfaction by improving service quality.

## 2. Materials and Methods

### 2.1. Reporting Source and Information Search Strategy

A systematic review and meta-analysis was performed, and their results were reported following the PRISMA (preferred reporting items for systematic reviews and meta-analyses) checklist; the study was registered on the International Prospective Register of Systematic Reviews (PROSPERO 2023 (CRD 42023424342).

Searches were conducted in several electronic databases, including Scopus, Web of Science, EMBASE, Medline/PubMed, Google Scholar, African Journals Online, and Semantic Scholar. These databases cover a wide range of academic and scientific literature, providing a diverse collection of articles. A search strategy was developed using Boolean operators (AND, OR) to combine and refine search terms. The specific search terms used in PubMed included keywords related to maternal satisfaction, children's vaccination, and associated factors. This strategy helps narrow the results down to articles that are most relevant to the research question. The search was limited to quantitative articles written in English. This restriction helps to maintain consistency in the language of the articles and ensures that the included studies can be easily analyzed and compared. In addition to the electronic database search, a manual search of the bibliographies of relevant articles was carried out. This approach involves examining the reference lists of identified articles to find additional sources that may not have been captured in the initial electronic search. It helps ensure a comprehensive review of the available literature. The grey literature deposited on university websites and online repositories was also searched. The search terms used in PubMed: (((((((((((((((((“maternal satisfaction”) OR (women satisfaction [Title/abstract])) OR (mothers' satisfaction [Title/abstract])) OR (caregiver satisfaction [Title/abstract])) OR (parents' satisfaction [Title/abstract])) OR (satisfaction [Title/abstract]) AND (children's vaccination)) OR (childhood immunization [Title/abstract])) OR (childhood vaccination [Title/abstract])) AND (predictors“)) OR (“risk factors”)) OR (“determinate”)) OR (“contributing factors”)) OR (“associated factors”)) AND (Ethiopia).

### 2.2. According to Our Criteria, Studies Were Included If They Met the Following Requirements

Study design: Among the studies covered in the search were cross-sectional, cohort, and case-control studies.

Study setting: All regions in Ethiopia.

Participants: Mothers of children were studied in this systematic study.

Publication status: Data from all published publications as well as gray literature.

Date of publication: All dates were taken into consideration.

Language of the published article: In our study, we included studies written in English.

### 2.3. Exclusion Criteria and Inclusion Criteria

In addition, research studies were ruled out if they met any of the following criteria: studies that have not been translated into English, or if the full text of articles is unavailable, other than maternal satisfaction with vaccination, editorial comments, conference proceedings, and qualitatively described works.

### 2.4. Quality Assessment Tool

To determine the quality of the study, two appraisers (NT and KA) independently evaluated it using the Joanna Briggs Institute Meta-analysis of Statistics Assessment and Review Tool (JBI-MAStARI) [28]. We used the JBI critical appraisal checklist tool, which includes nine criteria for cross-sectional studies. (1) Was the sample representative of the target population? (2) Were the study participants recruited in a suitable way? (3) Was it of adequate size? (4) Did the study subjects and the setting receive detailed descriptions? (5) Did the data analysis cover a sufficient number of samples? (6) Were objective standard criteria used for the measurement of the condition? (7) Was the condition reliably measured? (8) Was there an appropriate statistical analysis? (9) Are all important confounding factors/subgroups/differences identified and accounted for? (10) Were subpopulations identified using objective criteria? And if not, was the low response rate managed appropriately? Each answer ranged from 0 to 10 with 0 representing “*No*” and 1 representing “*Yes*.” The first four items assess selection bias and nonresponse bias, the next five to nine assess measurement bias, and the final item assesses analysis bias. Each element was assessed as a low or significant risk of bias. The overall risk of bias was defined based on the score of items of high risk of bias per study: low (2), moderate (3, 4), and high (5).

### 2.5. Data Extraction Tool and Process

Reviewing abstracts and full-text articles independently by two authors (NTA and KAA), using a preexisting data abstraction tool. The data extracted included the author's name, the country where the study was conducted, the design and setting of the study, its year of completion, and its sample size. Disagreements were resolved through conversation or repeating the process. Using Excel 2007 in Microsoft Office to store and manage the extracted data is a common practice due to its spreadsheet capabilities and ease of use.

### 2.6. Measurement of Results

The cut-off point of the demarcation threshold formula was used to categorize the overall satisfaction of caregivers into two categories: satisfied and unsatisfied: ((Total highest score–Total lowest score)/2 + Total lowest score). When a caregiver's score fell below the cut-off point, they were classified as “*unsatisfied*,” and those whose scores were more than or equal to the cut-off point were classified as “*satisfied*” [29, 30].

### 2.7. Statistical Analysis

To estimate the prevalence of maternal satisfaction with the child's vaccination service, the weighted inverse variance random-effects model [31] was employed. A subgroup analysis was implemented to adjust the variation in the pooled prevalence estimates according to the region in which the included studies were conducted. *I*^2^ statistics denoted low, moderate, and high heterogeneity between studies when the value denoted 25%, 50%, and 75%, respectively [32]. To assess publication bias, the funnel plot and Egger's regression test were used, and for adjustment, trim-and-fill analysis was conducted [33]. STATA Version 14 statistical software was used for meta-analysis.

## 3. Results

### 3.1. Search Results and Study Characteristics

**Figure 1:**
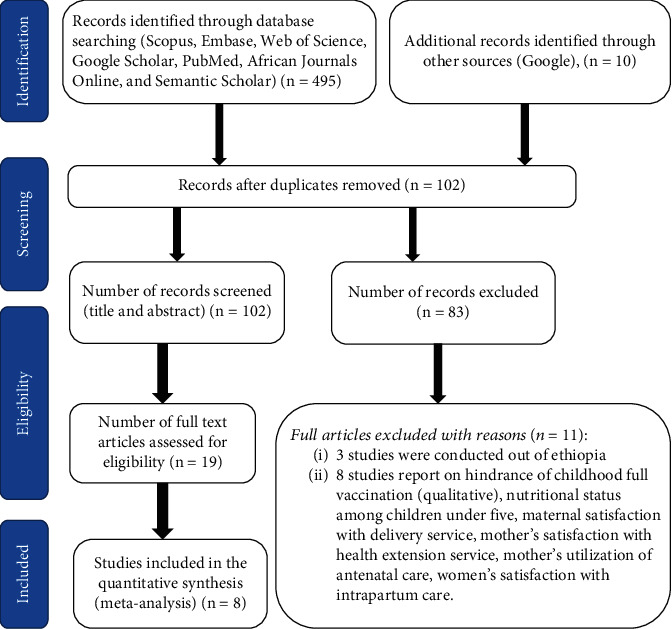
PRISMA flow diagram for the identification and selection of articles for this meta-analysis and systematic review.

Five hundred and five original article records were found through the online search of Scopus, Embase, Web of Science, Google Scholar, PubMed, African Journals Online, and Semantic Scholar, of which 403 duplicate records were removed. Out of 102 articles screened for titles and abstracts, 83 articles were excluded as irrelevant. Then, a total of 19 articles were reviewed in full text. Furthermore, 11 articles were excluded based on predetermined eligibility criteria. These are research studies focused on maternal satisfaction with the health extension service, qualitative synthesis on vaccination uptake, satisfaction of antenatal care, partial vaccination, preeclampsia, and postpartum intrauterine contraceptives, among other topics. Finally, eight articles were included in the meta-analysis (Figure 1).

### 3.2. Characteristics of the Included Studies

Eight studies were conducted in Ethiopia between 2018 and 2022. Of these, three were in the Southern Nations, Nationalities, and People's Region (SNNPs) [14, 25, 34], four in Amhara [1, 9, 26, 27], and one in Oromia [24]. All studies accessed through the search were cross-sectional. Sample sizes ranged from 382 (37) to 722 (41) (Table 1).

### 3.3. Quality of Included Studies

In all cross-sectional analyses, no methodological defects or significant bias were detected using the JBI Critical Assessment Checklist (Table 2).

### 3.4. Meta-Analysis

#### 3.4.1. The Prevalence of Maternal Satisfaction With Vaccination of Children in Ethiopia

**Figure 2:**
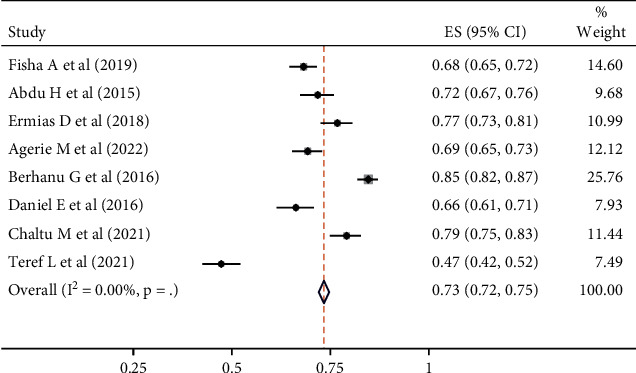
Forest plot displaying pooled estimates (ES) of maternal satisfaction with children's vaccinations. The midpoint and length of each segment represent the prevalence and 95% confidence interval (CI), while the diamond shape illustrates the combined prevalence across all studies.

The combined prevalence of maternal satisfaction with vaccination of children was found to be 73% (95% CI: 72–75; *I*^2^ = 0.00%, *p* value < 0.001) (Figure 2).

#### 3.4.2. Subgroup Analysis

**Figure 3:**
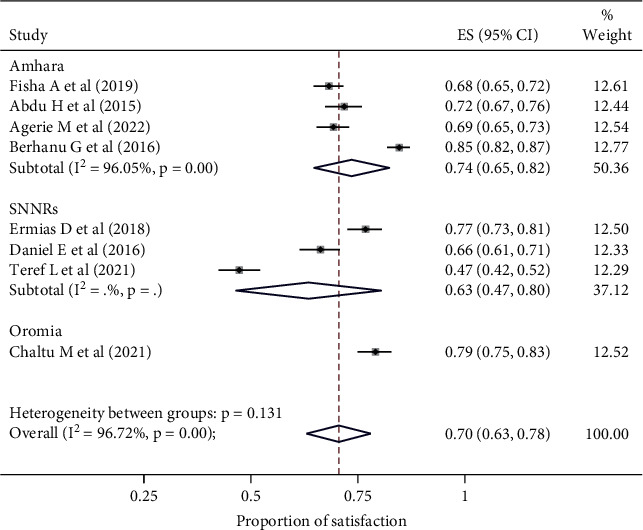
The forest plot showed the subgroup analysis of the prevalence (ES) of satisfaction of mothers with their children's vaccination. The midpoint and the length of each segment indicated a prevalence of each region and a 95% CI, while the diamond shape showed the combined prevalence of studies in each region.

Subgroup analysis based on the region where the studies were conducted. The result revealed that the prevalence of maternal satisfaction with vaccination of children was 63% in SNNPR, 79% in Oromia, and 74% in Amhara. (Figure 3).

#### 3.4.3. Sensitivity Analysis

**Figure 4:**
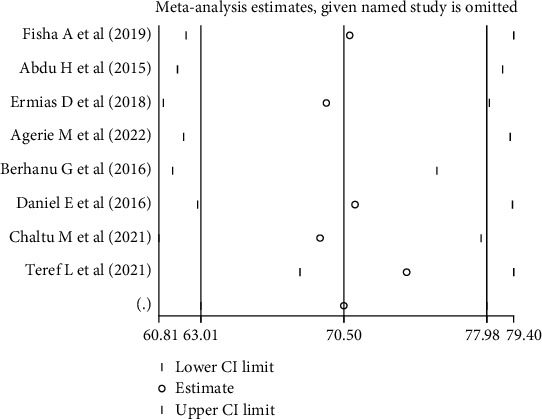
Sensitivity analysis of the prevalence of satisfaction of mothers with their children's vaccination in Ethiopia.

The sensitivity analysis showed that there was little change that could not affect the overall outcome estimate too much (Figure 4).

#### 3.4.4. Publication Bias

**Figure 5:**
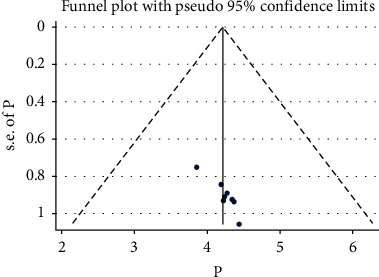
The funnel plot vertical lines estimate the effect size, whereas a diagonal line measures the precision of individual studies with a 95% confidence limit.

A funnel graph was used to see the symmetry of the publications (Figure 5), and the Egger test did not show publication bias (*p* value = 0.404).

#### 3.4.5. Test for Funnel Plot Asymmetry (Egger Test)

**Figure 6:**
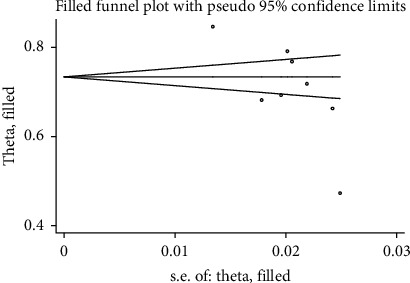
Funnel plot fill and trim analysis of satisfaction of mothers with their children's vaccination in Ethiopia.

There was statistical publication bias as measured by Egger's tests with *p* = 0.02. Additionally, a filled funnel trim analysis was performed to further investigate publication bias, but no studies were found to be significantly biased (Figure 6 and Table 3).

The estimated bias coefficient (intercept) is −20.50 with a standard error of 6.78, giving a *p* value of 0.02.

## 4. Determinant Factors Associated With the Satisfaction of Mothers With Their Children's Vaccination

### 4.1. Waiting Time

**Figure 7:**
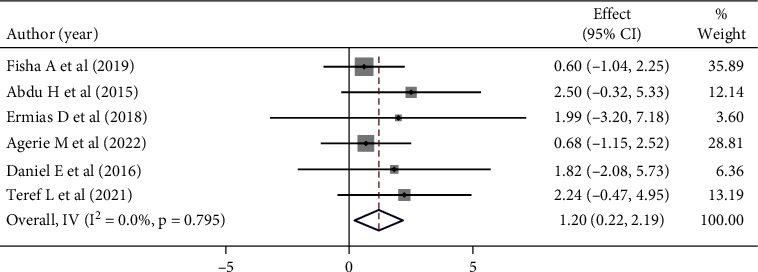
Forest plot showing the association between maternal satisfaction with their children's vaccination service and waiting time.

Mothers/caregivers who got a vaccination service within a short waiting time were 1.2 times more likely to be satisfied with the vaccination service that exists for children in health facilities than their counterparts. However, this was not statistically significant (OR: 1.20 (95% CI (0.22, 2.19), *I*^2^:0.0%. According to the heterogeneity test (*p* = 0.795), no significant variation was found between studies. Egger's test did not detect statistically significant publication bias (*p* = 0.089) (Figure 7).

### 4.2. Age of the Mothers

**Figure 8:**
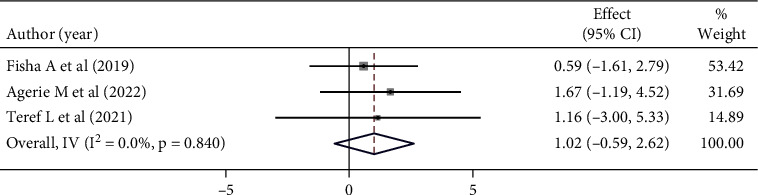
Forest plot showing the association between maternal satisfaction with their children's vaccination service and the age of the mothers.

Mothers/caregivers whose ages between 20 and 24 years were 1.02 times more likely to be satisfied with the child's vaccination service than the counterpart; however, this was not statistically significant (OR: 1.02 (95% CI (0.22, 2.19), *I*^2^:0.0%. Based on the heterogeneity test (*p* = 0.216), no significant variation was found between studies. Egger's test did not detect a statistically significant publication bias (*p* = 0.635) (Figure 8).

### 4.3. Vaccine Information

**Figure 9:**
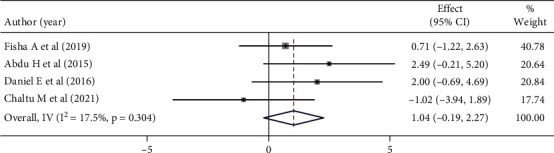
Forest plot showing the association between maternal satisfaction with their children's vaccination service and vaccination information.

Mothers/caregivers who received current vaccine information were 1.04 times more likely to be satisfied compared to those who did not receive current vaccine information. However, this was not statistically significant (OR: 1.04 (95% CI (−0.19, 2.27), *I*^2^:17.5%. Based on the heterogeneity test (*p* = 0.304), no significant variation was found between studies. Egger's test did not detect a statistically significant publication bias (*p* = 0.097). (Figure 9).

### 4.4. Maternal Knowledge

**Figure 10:**
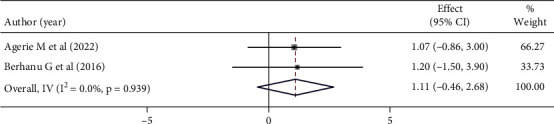
Forest plot showing the association between maternal satisfaction with their children's vaccination service and maternal knowledge.

Mothers who had good knowledge of vaccination had 11% higher chance of being satisfied than those mothers who did not know of vaccination. However, this was not statistically significant (OR: 1.11 (95% CI (−0.46,2.68), *I*^2^:0.0%. Based on the heterogeneity test (*p* = 0.939), no significant variation was found between studies (Figure 10).

### 4.5. Maternal Attitude

**Figure 11:**
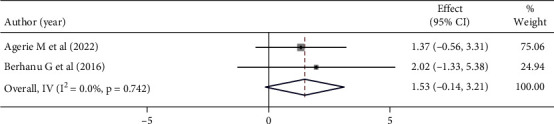
Forest plot showing the association between maternal satisfaction with their children's vaccination service and maternal attitude.

Mothers who had a favorable attitude toward childhood vaccination (AOR:1.53, 95% CI (−0.14–3.21), *I*^2^:0.0%) were nearly two times more likely to be satisfied than the reference group. However, this was not statistically significant. Based on the heterogeneity test (*p* = 0.742), no significant variation was found between studies (Figure 11).

### 4.6. Greetings

**Figure 12:**
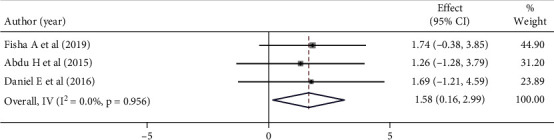
Forest plot showing the association between maternal satisfaction with their children's vaccination service and greetings.

Mothers who received greetings from the healthcare provider had a 58% higher chance of being satisfied compared to the reference group, although the difference was not statistically significant (OR: 1.58 (95% CI (0.16, 2.99), *I*^2^ = 0.0). The heterogeneity test did not produce significant differences between studies (*p* = 0.956) (Figure 12).

## 5. Discussion

Vaccination programs have been successful in preventing numerous diseases and reducing child mortality rates. However, there are still challenges that need to be addressed. These include hesitancy in vaccination [35], geographical obstacles [36], inadequate health infrastructure or financial restrictions [37], proper storage and transportation conditions [38], cultural beliefs, religious beliefs and social norms [39], insufficient health staff [37, 40], lack of training, and inadequate follow-up mechanisms [37]. Addressing these challenges requires a multifaceted approach involving public health education, community engagement, and improved access to vaccines, strengthening healthcare systems, and addressing vaccine hesitancy through evidence–based communication strategies. Governments, healthcare providers, researchers, and communities need to work together to ensure the success of vaccination programs and improve maternal satisfaction.

The finding of this study showed that the overall satisfaction with the service of mothers/caregivers with childhood vaccination was 73% (95% CI: 72–75; *I*^2^ = 0.00%, *p* value < 0.001). This finding is consistent with studies done in Iraq (73.2%) [41] and Egypt (75%) [42]. However, this study finding is a higher prevalence than that found in Nigeria (19.5%) [43], Vietnam (17.1%) [44], Pakistan (62%) [45], and South Nigeria (43.6%) [46]. This may be the result of differences in cultural beliefs, social norms, and socioeconomic factors that influence maternal attitudes toward vaccination. Among some societies, immunization can be viewed as a more important part of child health, resulting in greater satisfaction with the program. Furthermore, socioeconomic factors, such as income and education, can have a significant impact on access to healthcare and vaccination services and consequently affect satisfaction with these services. It is also possible to justify this claim by pointing out that healthcare infrastructure can vary from one country to another in terms of quality and accessibility. The availability of well-equipped healthcare facilities, including vaccination centers, may be lacking in some parts of Nigeria, Pakistan, and Vietnam. Having inadequate infrastructure can lead to limited availability of vaccines, long waiting times, or inadequate healthcare services, all of which can negatively affect mother satisfaction.

On the contrary, the finding of this study is lower than that found in Switzerland (89%) [47], United States (96%) [48], Egypt (95.2%) [49], Saudi Arabia (87.4%) [50], Nigeria (95.9%) [44], India (94%) [51], and Iraq (87%) [52]. This may be the reason for the differences in the quality and accessibility of healthcare infrastructure that can significantly impact maternal satisfaction. Developed countries such as the United States, Switzerland, and Saudi Arabia generally have well-established healthcare systems with a robust network of health facilities, including vaccination centers. In contrast, developing countries such as Ethiopia may face challenges related to inadequate healthcare infrastructure, including limited access to quality healthcare services and vaccine distribution centers [53, 54]. Insufficient infrastructure can contribute to lower maternal satisfaction. Another argument that could be made is that maternal satisfaction with vaccination can be influenced by the level of awareness and education about the importance of immunization [55]. Developed countries often have well-funded public health campaigns, comprehensive health education programs [56, 57], and effective communication strategies to promote awareness and understanding of vaccines among mothers [58, 59]. In contrast, developing countries may have limited resources for public health campaigns and health education initiatives, leading to a lower awareness and understanding of vaccines among mothers [37, 60].

Subgroup analysis shows a similar prevalence of maternal satisfaction with child vaccination in Oromia (79%) and the Amhara region (74%). This suggests that there may be certain factors or characteristics that are common between these two regions, contributing to comparable levels of maternal satisfaction. Here are some possible reasons for this similarity: The Oromia and Amhara regions in Ethiopia share similarities in terms of culture, traditions, and socioeconomic conditions. Cultural beliefs and social norms related to vaccination may be relatively consistent between the two regions, resulting in similar levels of maternal satisfaction. Additionally, both Oromia and Amhara regions have relatively equitable access to healthcare facilities, vaccination centers, and healthcare providers, so mothers in both regions may experience similar levels of satisfaction. If the vaccine coverage rates and vaccine availability are comparable in the Oromia and Amhara regions, it can contribute to similar levels of maternal satisfaction. In addition, both regions might face similar challenges or successes in terms of vaccine supply and distribution, which may result in comparable satisfaction rates. Similarities in healthcare infrastructure, including the presence of well-equipped healthcare facilities and trained healthcare professionals, can also contribute to comparable levels of maternal satisfaction.

The higher prevalence of maternal satisfaction with child vaccination in the Oromia and Amhara regions compared to the SNNPR in Ethiopia can be attributed to several potential factors. This could be because variations in healthcare infrastructure across regions can impact maternal satisfaction. The Oromia and Amhara regions may have better developed health systems with better access to health facilities, including vaccination centers. On the contrary, the SNNPR region may have relatively limited healthcare infrastructure, resulting in lower satisfaction rates. Additionally, both regions might have better access to vaccines and vaccination centers, including rural and remote areas, which can lead to higher satisfaction rates. On the contrary, the SNNPR region may face challenges related to the limited availability and accessibility of vaccines, contributing to lower satisfaction levels.

The results of this study demonstrated that several factors, including mother of age, waiting time, vaccination information, maternal attitude, knowledge, and greetings, significantly influenced how satisfied mothers were with the children's vaccination service. Therefore, compared to their counterpart, women who had short wait times (30 min) were more likely to be satisfied with the service. Similar findings were reported from a study done in Nigeria [61], India [51], Vietnam [62], and Guatemala [63]. It may be explained by the fact that short waiting times make it more convenient for mothers and caregivers to access vaccination services. Waiting for extended periods can be challenging, especially for those with other responsibilities or time constraints. When vaccination services are efficient and waiting times are minimized, it reduces the burden on mothers and improves their overall satisfaction with the process. However, long waiting times can cause increased stress and anxiety for mothers, especially when they have young children. When vaccination services are organized and waiting times are short, it alleviates stress and anxiety, creating a more positive experience for mothers and their children. This positive experience can improve maternal satisfaction with the vaccination process. In addition, short waiting times are often associated with efficient and well-organized healthcare services. When mothers experience minimal waiting times, it can give them the perception that the healthcare facility values their time and provides high-quality care. This positive perception can positively influence maternal satisfaction with the vaccination service and the overall health facility. In addition, short waiting times can improve compliance with vaccination schedules and increase vaccine coverage rates. When mothers have a positive experience with short waiting times, they are more likely to return for subsequent vaccinations and ensure that their children receive the recommended vaccines. Increased compliance and vaccine coverage contribute to improved health outcomes and can further improve maternal satisfaction.

The present study shows that the chances of having an increased level of satisfaction with the service were higher among mothers whose age lies between 20 and 24 years. This study finding is supported by the study report from China [64] and Iraq [41]. The possible justification is that young mothers, particularly those in their childbearing years, may have more flexibility in terms of their schedule and availability to take their children for vaccinations. They may have fewer competing responsibilities, such as work or other caregiving obligations, which can make it easier for them to access vaccination services. This convenience and ease of access can contribute to higher satisfaction levels. Some extra explanation that might be plausible is that young mothers, especially first-time mothers, may be more health-conscious and proactive when it comes to their children's well-being. They can actively seek vaccination services and stay informed about the importance of immunizations. This increased awareness and participation can lead to increased satisfaction with the vaccination service.

Compared to those who did not receive current vaccine information, mothers who did receive it were one time more likely to be satisfied. This could be likely because vaccine information helps mothers understand the importance of immunizing their children against vaccine-preventable diseases. When they are aware of the potential risks and complications associated with these illnesses, they can feel more confident in their decision to vaccinate their children. The knowledge that vaccines can protect their children from potentially severe or life-threatening diseases improves their satisfaction in ensuring their children's health and well-being. The other probable explanation is that vaccine information can alleviate concerns and anxieties mothers may have about potential vaccine side effects or risks. When they have access to accurate information on the safety profile of vaccines and the rigorous testing and monitoring processes involved, they can make informed decisions with peace of mind. This reassurance can significantly contribute to maternal satisfaction in ensuring their children's health and safety.

Parents who are knowledgeable about vaccinations have been shown to be more likely to be satisfied with vaccination services. The same issues have been observed in other countries, such as Kenya [65], Sub-Saharan Africa [37], Burkina Faso [66], Nigeria [67], and Angola [68]. Here are a few possible explanations: When parents have a good understanding of vaccines, including their benefits, risks, and importance, they can make informed decisions about vaccinating their children. This knowledge allows them to feel confident in their choices and increases their satisfaction with vaccination services. Additionally, parents who are knowledgeable about vaccinations can engage in more effective communication with healthcare providers. They can ask informed questions, seek clarification, and have meaningful discussions about vaccines. This open and productive communication improves their satisfaction with the vaccination services they receive. On the contrary, mothers' satisfaction with childhood immunization services and knowledge was not statistically significant [49]. Several factors may contribute to this, including differences in methodologies, sample sizes, and populations.

Having a positive attitude toward children's vaccinations is one of the predictors of mothers' satisfaction with their healthcare services. It is in agreement with studies conducted previously been conducted in Nigeria [67], Georgia [69], and Thailand [70]. This could be related to the fact that having a positive attitude toward vaccinations can alleviate concerns and anxieties that mothers may have about their children's health. They feel reassured that by vaccinating their children, they are providing them with added protection against vaccine–preventable diseases. This peace of mind improves maternal satisfaction. In addition, a positive attitude toward vaccinations often indicates a level of trust in healthcare providers who recommend and administer vaccines. When mothers have confidence in the expertise and guidance of healthcare professionals, it enhances their satisfaction with the vaccination services they receive.

The results of this study indicate that mothers who received greetings had a higher probability of being more satisfied with the service. The associations were reported in previous studies like Nigeria [71], Australia [72], and Colombia [73]. A possible justification that has the potential to be raised is that greetings can convey care, empathy, and support. For mothers who may be anxious or stressed about their child's healthcare visit, receiving a friendly greeting can help alleviate those emotions. Feeling emotionally supported contributes to a positive service experience and improves satisfaction. The other probable explanation is that when mothers are greeted warmly and respectfully by healthcare providers or staff, it helps establish a positive rapport and a welcoming atmosphere. This personal touch improves the overall experience and contributes to increased satisfaction with the service received.

## 6. Strengths and Limitations of This Study

This systematic review and meta-analysis on mothers' child vaccination in Ethiopia demonstrates some strengths, including rigorous design, comprehensive evidence synthesis, and implications for policymaking and intervention implementation. These strengths enhance the validity and relevance of the study. As a result of these strengths, the study becomes a valuable resource for researchers, policymakers, and healthcare practitioners who are involved in vaccination programs in Ethiopia.

Studies included in a systematic review and meta-analysis may have variations in study design, participant characteristics, interventions, outcomes, and other factors. This heterogeneity can limit the ability to combine the results into a meaningful summary estimate. Statistical methods, such as random-effects models, are often used to account for heterogeneity, but they may still impact the interpretation of the findings. Furthermore, the limitations of individual studies included in a systematic review or meta-analysis can also affect the overall quality and reliability of the findings.

## 7. Conclusion

A meta-analysis of mothers' satisfaction with vaccination services for their children in Ethiopia found a low level of satisfaction. Therefore, provide regular training and capacity-building programs for healthcare workers involved in the delivery of vaccination services. Use various communication channels, including the media, community health workers, and local community leaders, to distribute accurate and culturally sensitive information. Develop robust monitoring and evaluation systems to assess the quality, coverage, and satisfaction of vaccination services.

## Figures and Tables

**Table 1:** Characteristics of articles included in the systematic review and meta-analysis (*n* = 8).

**Author (year)**	**Study area**	**Study design**	**Sample size**	**Prevalence of maternal satisfaction on vaccination (95% CI)**	**Quality**
Fisha et al. (2019)	Amhara	Community–based cross-sectional study	682	68.2%	Low risk
Abdu et al. (2015)	Amhara	Facility–based cross-sectional study	422	71.9%	Low risk
Ermias et al. (2018)	SNNRs	Facility–based cross-sectional study	422	76.7%	Low risk
Agerie et al. (2022)	Amhara	Facility–based cross-sectional study	554	69.3	Low risk
Berhanu et al. (2016)	Amhara	Community–based cross-sectional study	722	84.6%	Low risk
Daniel et al. (2016)	SNNRs	Facility–based cross-sectional study	382	66.2%	Low risk
Chaltu et al. (2021)	Oromia	Facility–based cross-sectional study	407	79.1%	Low risk
Teref et al. (2021)	SNNRs	Facility–based cross-sectional study	402	47.2%	Low risk

**Table 2:** The Joanna Briggs Institute (JBI) Prevalence Critical Appraisal Tool.

**s/n**	**Criteria**	**Yes**	**No**	**Unclear**	**Not applicable**
1.	Was the sample representative of the target population?	✓			
2.	Were study participants recruited in an appropriate way?	✓			
3.	Was the sample size adequate?	✓			
4.	Were the study subjects and the setting described in detail?	✓			
5.	Was the data analysis conducted with sufficient coverage of the identified sample?	✓			
6.	Were objective, standard criteria used for the measurement of the condition?	✓			
7.	Was the condition measured reliably?	✓			
8.	Was there an appropriate statistical analysis?	✓			
9.	Are all important confounding factors/subgroups/differences identified and accounted for?	✓			
10.	Were subpopulations identified using objective criteria?	✓			

**Table 3:** Test for asymmetry in funnel plot (Egger's test).

**Number of ** **s** **t** **u** **d** **i** **e** **s** ** = 8**					**Root ** **M** **S** **E** ** = 3.752**	
**Std_eff**	**Coef.**	**Std. err.**	**t**	**p** **>** ***t***	**(95% conf. interval)**	
slope	1.121218	0.1306695	8.58	0.000	0.8014814	1.440955
bias	−20.50314	6.780191	−3.02	0.023	−37.09367	−3.91261

*Note:* Test of H0: no small-study effects *p* = 0.023.

## Data Availability

All data are available in the paper.

## Nomenclature

AJO: Africa Journal Online
CI: confidence interval
ES: effect size
JBI: Joanna Briggs Institute
OR: odds ratio
PRISMA: preferred reporting items for systematic review and meta-analysis
SNNPR: South Nations, Nationalities, and People's Region
